# Biphenyl-Bridged Organosilica as a Precursor for Mesoporous Silicon Oxycarbide and Its Application in Lithium and Sodium Ion Batteries

**DOI:** 10.3390/nano9050754

**Published:** 2019-05-16

**Authors:** Manuel Weinberger, Po-Hua Su, Herwig Peterlik, Mika Lindén, Margret Wohlfahrt-Mehrens

**Affiliations:** 1 Helmholtz Institute Ulm (HIU), Karlsruher Insitute of Technology, Helmholtzstraße 11, D-89081 Ulm, Germany; surobert82803@gmail.com (P.-H.S.); Margret.Wohlfaht-Mehrens@zsw-bw.de (M.W.-M.); 2 Faculty of Physics, University of Vienna, Boltzmanngasse 5, A-1090 Vienna, Austria; herwig.peterlik@univie.ac.at; 3 Insitute for Inorganic Chemistry II, Ulm University, Albert-Einstein-Allee 11, D-89081 Ulm, Germany; mika.linden@uni-ulm.de; 4 Zentrum für Sonnenenergie-und Wasserstoffforschung (ZSW), Helmholtzstraße 8, D-89081 Ulm, Germany

**Keywords:** organosilica, mesoporous, silicon oxycarbide, sodium ion battery, lithium ion battery

## Abstract

Silicon oxycarbides (SiOC) are an interesting alternative to state-of-the-art lithium battery anode materials, such as graphite, due to potentially higher capacities and rate capabilities. Recently, it was also shown that this class of materials shows great prospects towards sodium ion batteries. Yet, bulk SiOCs are still severely restricted with regard to their electrochemical performance. In the course of this work, a novel and facile strategy towards the synthesis of mesoporous and carbon-rich SiOC will be presented. To achieve this goal, 4,4′-bis(triethoxysilyl)-1,1′-biphenyl was sol–gel processed in the presence of the triblock copolymer Pluronic P123. After the removal of the surfactant using Soxhlet extraction the organosilica material was subsequently carbonized under an inert gas atmosphere at 1000 °C. The resulting black powder was able to maintain all structural features and the porosity of the initial organosilica precursor making it an interesting candidate as an anode material for both sodium and lithium ion batteries. To get a detailed insight into the electrochemical properties of the novel material in the respective battery systems, electrodes from the nanostructured SiOC were studied in half-cells with galvanostatic charge/discharge measurements. It will be shown that nanostructuring of SiOC is a viable strategy in order to outperform commercially applied competitors.

## 1. Introduction

There is an urgent need to reduce greenhouse gas emissions significantly to lower the aftermath of the anthropogenic climate change. With the transition to a renewable and sustainable energy infrastructure, batteries will become more and more important, for instance, in order to store energy spillover or to power electric vehicles. Lithium ion batteries based on intercalation chemistry currently represent the state-of-the-art, however, this technology is limited to energy densities of around 250 Whkg^−1^ on a full cell level. Electrode materials utilizing alloying or a conversion-based reaction throughout battery operation may exceed energy densities of conventional battery active materials, still, much effort has to be invested to deal with other problems, such as huge volume expansion or poor coulombic efficiencies. In addition, lithium is a rather limited resource, which might at some point lead to a shortage, and thus increased cost. Recently, other so-called post lithium battery technologies, such as sodium ion batteries, are gaining increasing attention.

On the anode side, graphite is still the material of choice. It offers the advantage of small volume expansion during lithiation, which leads to superior cycle life. However, its capacity is rather limited with 372 mAhg^−1^ and its rate capability is generally poor due to slow intercalation kinetics. In addition, it is practically inactive towards sodiation [1]. More disordered carbon materials such as hard carbons generally show slightly lower capacities in the lithium system, but significantly outperform graphite in the case of sodium. Sodiation capacities of up to 300 mAhg^−1^ at lower C-rates of C/80 were reported by Stevens and Dahn [2]. A possible explanation lies in a larger graphite interlayer spacing, leading to facilitated intercalation of the larger sodium ions. This is also supported by findings of Wen et al. on the application of expanded graphites as sodium ion battery anodes [3]. However, higher capacities at higher specific currents may only be achieved when utilizing nanoscopic carbon materials due to shorter diffusion pathways. Hu et al. synthesized hierarchically porous carbon monoliths via nanocasting with mesophase pitch and subsequent carbonization at 700 °C. When cycled versus lithium, the resulting material was able to retain capacities of around 250 mAhg^−1^ at 10C and when increasing the current to 50C still a 100 mAhg^−1^ could be obtained [4]. Wenzel et al. were the first to prove this concept with a similar carbon material in the case of sodium ion batteries [5]. Although the reversible capacities at a rate of C/5 were relatively small, with around 130 mAhg^−1^, capacities of 100 mAhg^−1^ could still be achieved at 5C. Tang et al. cycled hollow carbon nanospheres versus sodium [6]. At a lower current of 100 mAg^−1^ around 200 mAhg^−1^ were obtained and at 2 Ag^−1^ 100 mAhg^−1^ could be retained.

Silicon oxycarbides (SiOC) are an interesting alternative to carbon- or alloy-based anode materials. These materials may be considered as hybrids consisting of intimately mixed phases of carbon and silica with covalent bonds between carbon and silicon in the interdomain boundary forming mixed [SiO_4−x_C_x_] species. Their synthesis is generally achieved by the pyrolysis of sol–gel derived organosilica materials or cross-linked silicones [7,8,9,10,11,12,13,14,15]. Based on the chemical composition of the precursor and the pyrolysis temperature it is possible to carefully tune the microstructure and the resulting chemical composition of the final materials. For electrochemical applications, higher contents of free carbon are usually desired in order to achieve higher conductivities. Especially preceramic precursors containing larger amounts of phenyl or phenylene moieties have previously shown to result in SiOCs with free carbon contents > 30 wt % [14,16,17]. SiOCs are able to deliver high capacities in the lithium system of well above 800 mAhg^−1^ [14,16,18] and at the same time show a significantly smaller volume expansion compared to, for instance, elemental silicon [14]. This was recently demonstrated by Halim et al., which investigated electrodes made of phenyl-rich silicon oil derived carbon-rich silicon oxycarbides. They found that, upon lithiation, the thickness of the electrode increased by only 36% whereas a silicon reference increased by 303% [14]. The higher capacities may not only be explained by the contribution of the free carbon phase, but must also originate from the ceramic part [19,20]. It is generally believed that lithium storage occurs (I) in interstitial spaces or edges of graphene layers of the free carbon phase, (II) that micropores make a minor contribution, and (III) that an even lower amount of the capacity may be attributed to conversion reactions of the different [SiC_x_O_4−x_] units within the covalent ceramic framework [18,19,20]. In addition, SiOCs may also be utilized as anode materials in sodium ion batteries. Only recently, we have shown that submicrometric SiOC spheres may provide capacities of up to 200 mAhg^−1^, which makes them interesting alternatives to other materials such as hard carbons and nanoscopic TiO_2_ [16,19,21].

To date, there is only a limited amount of reports on the direct thermal conversion of nanoporous organosilica materials into SiOC. The group from Babonneau was the first to take a deeper look into the conversion of periodic mesoporous organosilicas into SiOC with the aid of X-ray diffraction and solid-state nuclear magnetic resonance (NMR) techniques [22,23,24,25]. They utilized carbosilanes with smaller organic moieties, such as the ethane-bridged ones, for the synthesis of the organosilica precursor. Although it was possible to maintain the mesostructures up to higher temperatures of 1000 °C, only very small amounts of carbidic silicon species could be detected, what is most certainly attributed to the limited carbon content in the organosilica material and an unfavorable O/Si ratio. Decreasing the latter one, by for instance, utilizing a cyclic silane, such as a trisilacyclohexane-derivate, was shown to be more favorable for the formation of larger fractions of carbidic silicon species, also in the case of highly porous samples [7,26,27]. However, the free carbon content in such materials is relatively low, which is not beneficial for electrochemical applications. Organosilicas with phenyl or phenylene moieties are well known to produce SiOCs with large amounts of free carbon and also larger fractions of carbidic silicon species [SiO_4−x_C_x_] [14,16,19,21], however, their potential for the synthesis of carbon rich highly porous SiOC materials and thus interesting battery materials has not gained much attention yet.

In order to improve the electrochemical properties of SiOCs further, nanosizing or nanostructuring is a suitable strategy. Pradeep et al., for instance, reported on an approach to synthesize SiOC aerogels via the pyrolysis of a preceramic polymer derived by a cross-linking reaction of linear polyhydridomethyl siloxane with divinylbenzene, which showed dramatically enhanced rate capabilities in lithium ion batteries at specific currents of up to 3600 mAg^−1^ [17]. With the current work, we want to present a novel and alternative approach to synthesize highly nanoporous SiOC by direct pyrolysis of a mesoporous organosilica material obtained from sol–gel processing of 4,4′-bis(triethoxysilyl)-1,1′-biphenyl in the presence of the triblock copolymer Pluronic P123. The resulting SiOC material maintains the structural features and constitutes even slightly higher surface area and pore volume. In the course of this publication, the materials properties will be compared in detail to the initial organosilica material. To get a deeper insight into the electrochemical performance of the pyrolyzed sample as an anode material in either sodium and lithum ion batteries, galvanostatic charge/discharge measurements will be applied to derive gravimetric capacities, rate capability, and cycle stability.

## 2. Materials and Methods 

### 2.1. Synthesis of the Biphenyl-Bridged Silane Derived SiOC

The organosilica material (denoted as BPO) was synthesized according to a procedure described previously by Yang and Sayari [28]. 0.5 g of Pluronic P123 (Sigma-Aldrich, Saint Louis, MO, USA, Mn = 5800 gmol^−1^) were dissolved in 18 g of deionized water. To this solution, 0.98 g of aqueous hydrochloric acid (Merck, Darmstadt, Germany, 35 wt %) and 0.74 g of butanol Alfa Aesar, Ward Hill, MA, USA, 99.4+%) were added. The mixture was heated to 40 °C and 1.6 g of 4,4′-bis(triethoxysilyl)-1,1′-biphenyl (Sigma-Aldrich, Saint Louis, MO, USA, 95%) were slowly dropped into the solution using a syringe. The final molar ratio of Silane:P123:butanol:HCl:H_2_O was 1:0.012:1.16:1.22:130. The reaction was further stirred for a duration of 24 h. A white powder was received after filtration, which was subsequently treated with acetone in a Soxhlet extractor to remove the Pluronic P123 template. The organosilica material was dried at 60 °C for 24 h. Carbonization was performed within a modified CWF1100 oven from Carbolite under a flow of argon. The heating rate was set to 10 °C min^−1^ and the final temperature was kept for 2 h. Prior to heating, three evacuation/purging cycles were applied to completely replace air and moisture by inert argon gas. A black powder was obtained and the ceramic yield was determined to be 70%. The carbonized material is denoted as BPO-C.

### 2.2. Material Characterization

The morphology of the BPO and BPO-C materials was characterized by scanning electron microscopy (SEM), using a Zeiss Gemini microscope. Samples were coated with a 4 nm thin layer of Au. SEM images were recorded with an in-lens detector at an accelerating voltage of 3 kV and a working distance of 3 mm.

Transmission electron microscopy images were recorded on a ZEISS EM 109 microscope, operated at 80 kV accelerating voltage. Samples were prepared via microtomy to yield slices with a thickness of around 100 nm.

Small-angle X-ray scattering (SAXS) experiments were performed with Cu-K_α_ radiation (wavelength 0.1542 nm) from a microfocus source (Incoatec High Brilliance) using a pinhole camera system (Nanostar, Bruker AXS, equipped with a 2D position sensitive detector). Samples were placed between tapes, measured for 600s, radially integrated and background corrected to result in SAXS intensities in dependence on the scattering q in a q-range of 0.1 to 2.0 nm^−1^.

X-ray diffraction (XRD) using Cu-K_α_ radiation (λ = 0.154 nm) was performed on a PANalytical XPert Pro. 

Raman spectra were recorded on a WITec Alpha 300R microscope. An excitation wavelength of 633 nm was applied and a CCD detector and a ×100 objective with NA = 0.9 were used. 

The carbon content of the BPO-C was determined with a Vario Micro Cube from Elementar.

Nitrogen sorption measurements were performed at 77 K on a Quadrasorb SI (Quantachrome). BPO(-C) samples were outgassed at 150 °C prior to the measurements. The specific surface areas were determined by application of the Brunauer–Emmett–Teller (BET) equation in the lower relative pressure range (p/p_0_ = 0.05–0.15), where the BET plot behaves in a linear fashion. The pore volumina were determined at a relative pressure p/p_0_ of 0.95. Pore size distributions were derived from the adsorption branch using the Barret–Joyner–Halenda (BJH) method. 

### 2.3. Electrochemical Characterization

188 mg of BPO-C were mixed with 23.5 mg Timcal Super P conductive additive (Imerys Graphite & Carbon, Bironico, Switzerland). 400 mg of a mixture of 500 mg PVDF (MTI corp., Richmond, CA, USA) in 8 g N-methyl-2-pyrrolidone (NMP, Sigma-Aldrich, Saint Louis, MO, USA, anhydrous) was added to the powder. This results in a weight ratio of active material:Timcal:PVDF of 80:10:10. Further NMP was added until the desired viscosity of the slurry was achieved. The slurry was then applied on rough copper foil (Schlenk, Roth, Germany) using doctor blade technique with a wet film thickness of 150 microns, which resulted in a dry layer active material loading of 1.3 mgcm^−2^ (related solely to the mass of active material). Circular electrodes with a diameter of 12 mm were punched out and subsequently dried under vacuum at 100 °C overnight. CR2032 button cells were fabricated within an argon filled glovebox. A glass fiber filter paper (GF/A, Whatman) was used as a separator and either a lithium metal platelet (MTI Corp., Richmond, CA, USA) or pressed and punched pieces of metallic sodium (Acros Organics, Geel, Belgium, dry sticks) served as counter electrodes. For lithium, LiPF_6_ in a 50/50 (v/v) mixture of ethylene carbonate and dimethyl carbonate (UBE industries, Ube, Japan, battery grade) was applied. In the case of sodium, NaClO_4_ (Alfa Aesar, Ward Hill, MA, USA, anhydrous) dissolved in propylene carbonate (BASF, Ludwigshafen, Germany, Selectilyte) was used. A fixed amount of 100 µL of electrolyte was added to each button cell. Galvanostatic charge/discharge measurements were performed at a temperature of 25 °C using a VMP3 galvanostat from BioLogic Science Instruments. Rate capability measurements at specific currents of 50, 100, 200, 500, 1000, 2000, and 3000 mAg^−1^ were carried out for both alkali metals. After the rate capability measurements, the specific current was restored to 50 mAg^−1^ to test for cycle stability. Lower and upper cut-off potentials for lithium and sodium were set to 0.005 and 3 V for all measurements. 

## 3. Results and Discussion

**Figure 1:**
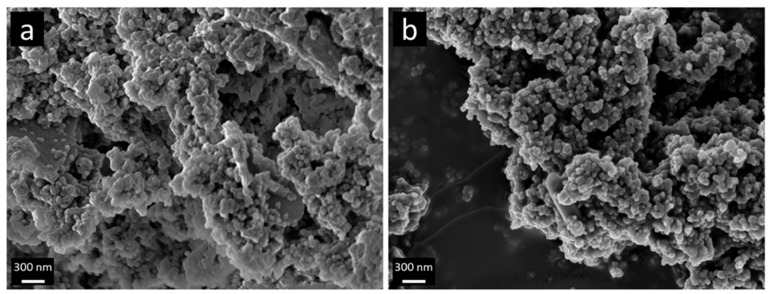
SEM images of samples (**a**) BPO and (**b**) BPO-C.

**Figure 2:**
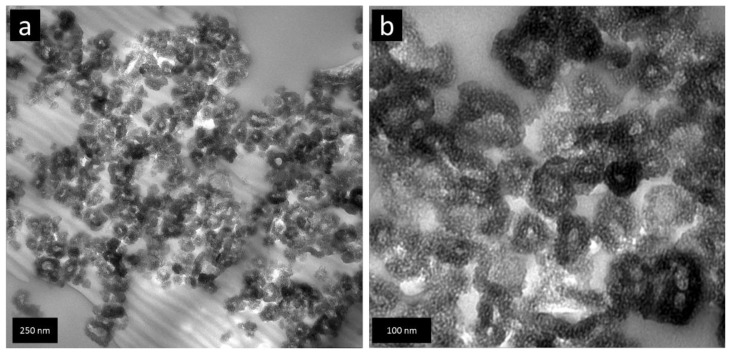
TEM images of sample BPO-C at low (**a**) and high (**b**) magnification.

In Figure 1a,b, SEM images for BPO and BPO-C are shown. The samples consist of aggregates of smaller particles with diameters of around 50–100 nm. Obviously, the shape and the particles dimensions may be retained upon thermal treatment at 1000 °C. To get a deeper insight into the structural features on the lower nanometer scale, TEM images of sample BPO-C were recorded, which are illustrated in Figure 2a,b. The measurements confirm the formation of particulate aggregates as were observed via SEM, however, interestingly, the particles are actually hollow spheres. It can be excluded that the hollow space is generated throughout carbonization, since the organosilica material shows similar particles (see Figure S1). A possible explanation might lie in the utilization of 1-butanol as an additive in the synthesis of the organosilica precursor. This compound is significantly more hydrophobic than water and thus might form an emulsion together with the biphenyl-bridged silane, which is probably further stabilized by the surfactant P123. Polycondensation then will occur on the surface of the emulsion droplets. A closer look at the hollow spheres in Figure 2b reveals significant amount of surfactant templated nanoporosity within the shells. A larger image may also be found in Figure S2.

**Figure 3:**
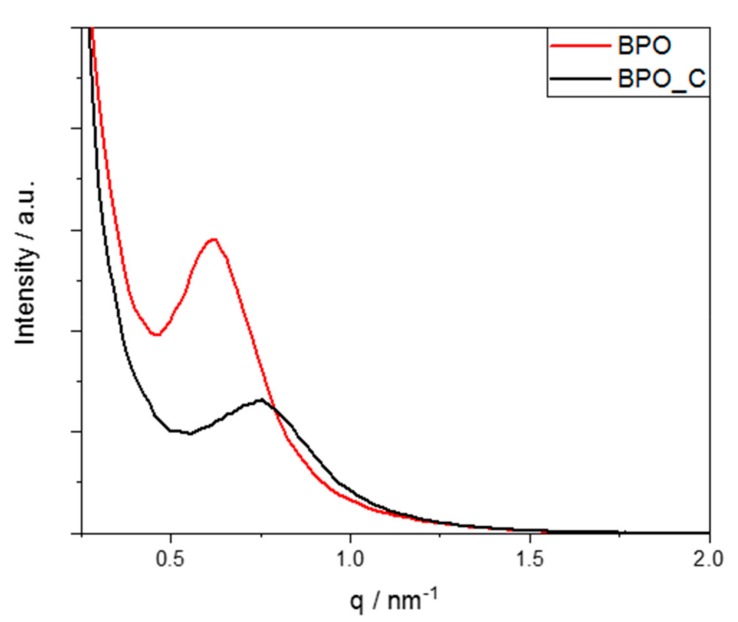
SAXS measurements for samples BPO and BPO-C.

Small-angle X-ray scattering was conducted to determine the formation of a possible mesostructure for samples BPO and BPO-C. In Figure 3, the corresponding scattering plots are illustrated. Both samples show a broad peak between 0.5 and 1 nm^−1^, corresponding to a typical periodicity of 4.6 and 3.8 nm for BPO and BPO-C, respectively. These values may directly be related to the mesopore sizes. Obviously, a slight shrinkage occurred due to the carbonization of the organosilica sample. Together with the observations from TEM, it may be concluded that a wormhole-like mesostructure was formed during the polycondensation reaction. 

**Figure 4:**
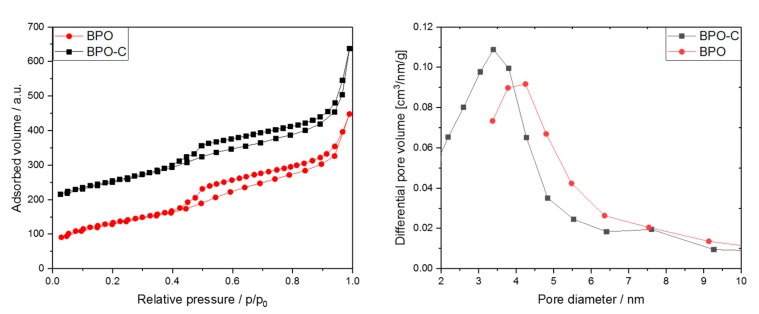
Sorption measurements for samples BPO and BPO-C. Sorption curves are shown in the left graph. The curves were separated by 100 cm^3^g^−1^ for clarity. BJH pore size distributions are illustrated in the right plot.

The nitrogen sorption isotherms in Figure 4a for both samples show adsorption over a relatively wide p/p_0_ range followed by a distinct condensation step at higher relative pressures due to condensation in interparticulate pores. The broad hysteresis is indicative of mesopores connected to the outside through smaller meso- or micropores. The desorption step at about 0.48 p/p_0_ is indicative of pore emptying due to cavitation [29]. The specific surface areas derived from BET plots for BPO and BPO-C amount to 462 m^2^g^−1^ and 527 m^2^g^−1^, respectively. The slightly higher surface area for BPO-C is most likely explained by the formation of a free carbon phase throughout carbonization. The same explanation may also be applied to a slightly increased pore volume of 0.55 cm^3^g^−1^ for BPO-C compared to 0.5 cm^3^g^−1^ for the organosilica sample. However, a larger pore volume may also be attributed to the occurrence of carbonization-induced formation of micropores in the case of BPO-C. In Figure 2b, the corresponding BJH pore size distributions calculated based on the adsorption branch are illustrated. For BPO, the maximum lies at a pore diameter of around 4.5 nm, whereas for BPO-C, the pore size distribution is shifted to smaller diameters, with the maximum centeredaround 3.5 nm. Sorption as well as SAXS data is also summarized in Table 1. 

**Figure 5:**
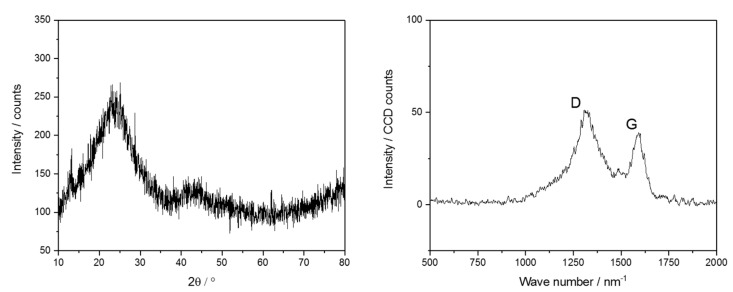
XRD plot (**left**) and Raman spectrum (**right**) for sample BPO-C.

Combustion-based elemental analysis revealed a carbon content of 45 wt %, what is a typical value for carbon-rich SiOC materials. This carbon is usually distributed between the ceramic SiOC phase and a free carbon phase. To further confirm the formation of the free carbon phase additional XRD and Raman spectroscopy measurements were performed. The results are presented in Figure 5. According to XRD the sample is amorphous showing a broad peak centered around 23° which is most likely associated to the [SiO_4−x_C_x_] units and free carbon. There is also a smaller peak at 43°. This one can be assigned to disordered carbon structures [30,31]. The Raman spectrum gives direct evidence for the formation of free carbon by the occurrence of the D-band, which is related to disorder-induced breathing motions of six-fold aromatic rings, and the G-band, which is induced by in-plane bond stretching of sp^2^ hybridized C atoms. The occurrence of such an intense disorder-induced D band together with a graphite G band (I_D_/I_G_ ratio of 2.9), with the latter one being shifted to values of 1600 cm^−1^, is direct evidence for a more disordered distribution of nanocrystalline graphite domains [12].

**Figure 6:**
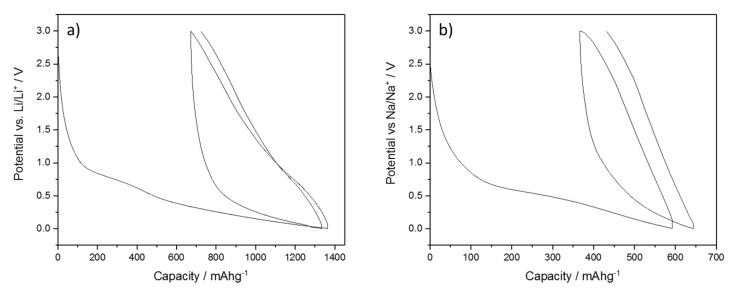
First two galvanostatic charge/discharge curves for BPO-C half-cell measurements against (**a**) lithium and (**b**) sodium. A specific current of 50 mAg^−1^ was used. Lower and upper cut-off potentials were set to 0.005 and 3 V, respectively. The active material loading was around 1.3 mgcm^−2^. As electrolyte, either 1 M LiPF_6_ in EC/DMC or 1 M NaClO_4_ in propylene carbonate were applied.

BPO-C was thoroughly tested as an anode material for lithium and sodium ion batteries. The galvanostatic charge/discharge curves with lower and upper cut-off potentials of 0.005 and 3 V for half cell measurements at a specific current of 50 mAg^−1^ against metallic lithium and sodium are shown in Figure 6. The curves show the typical characteristics for silicon oxycarbide samples. In the case of lithium, the first cycle shows a huge charge capacity of 1348 mAhg^−1^ which results in a coulombic efficiency of only 50%. This can be explained by the large surface area of the sample, which leads to increased solid electrolyte interface (SEI) formation (see broad shoulder between 100 and 600 mAhg^−1^). After SEI formation, the system shows stable cycling. It has to be noted that such low first cycle coulombic efficiencies render the material impractical for real world applications. However, in a recent publication, we have shown that prelithiation, for instance with lithium metal powders, is a great strategy to overcome this issue [15]. The initial discharge capacity is as high as 674 mAhg^−1^, almost twice as high as for graphite. Another obvious feature is the very broad hysteresis between charge and discharge what might also be explained by the larger surface area due to higher concentration of lithium ions in the SiOC/electrolyte interface [32]. In the case of sodium, the capacities are lower in general. It was recently shown that silicon shows indeed minor activity towards sodium [19], yet, the main fraction of the reversible capacity may most certainly be attributed to sodium storage into the free carbon phase. This is also supported by Fukui et al., who have argued that the main storage site for lithium is located in interstitial spaces or edges of the graphene layers of the free carbon phase [20]. Micropores and the conversion reaction of [SiC_x_O_4−x_] species play only a minor role in the process. Since sodium shows lower activity towards carbon in general, this might be a possible explanation for lower capacities of the carbon-rich SiOC. Due to the large surface area and the reduced electrochemical activity, the irreversible capacity in this case leads to an even lower first cycle efficiency of 38% with a first cycle discharge capacity of 226 mAhg^−1^, what is still a relatively high value for a sodium ion battery anode material. Capacities and efficiencies for the 1st and 10th cycle are also summarized in Table 2.

**Figure 7:**
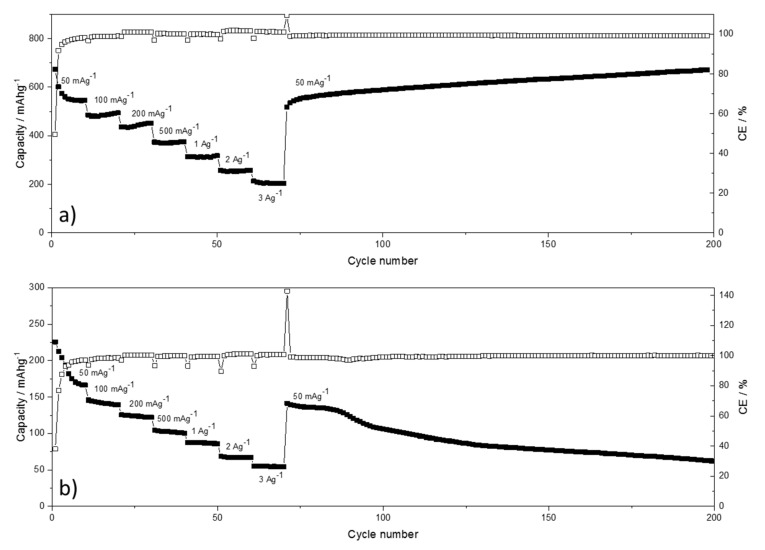
Rate capability and cycle stability plots BPO-C half-cell measurements against (**a**) lithium and (**b**) sodium. Lower and upper cut-off potentials were set to 0.005 and 3 V, respectively. The active material loading was around 1.3 mgcm^−2^.

The performance of BPO-C with regard to rate capability and cycle stability for both lithium and sodium are shown in Figure 7. BPO-C shows very good rate capability in general. In the lithium system still 200 mAhg^−1^ may be extracted at a very high specific current of 3Ag^−1^. After cycling at severe conditions, the system is able to recover the high capacities of around 600 mAhg^−1^ at 50 mAg^−1^. In fact, the capacities even increase slightly up to the 200th cycle. As the material experiences continuous cycles of expansion/contraction, silicon oxide/oxy carbide moieties, as well as free carbon structures might become progressively more accessible for lithiation/delithiation reactions. For sodium, the reduced diffusion path ways due to the nanoporosity seem to provide a beneficial effect as well. For instance, at 500 mAg^−1^, solid 100 mAhg^−1^ may still be extracted. Although the system shows a certain reversibility when returning to 50 mAg^−1^ after the rate capability measurement, the capacities start to decline continuously after around 90 cycles. Possible reasons for this behavior in contrast to the lithium system might lie on the one hand on the higher reactivities of sodium and perchlorate towards various cell components. In this course, also the higher cut-off potential of 3V has to be considered detrimental. On the other hand, the electrolyte itself is not a commercial product and has thus to be prepared in the lab with individual chemicals what might result in a lower performance.

## 4. Summary

Mesoporous carbon-rich silicon oxycarbide has been successfully synthesized by the pyrolysis of a mesoporous organosilica precusor which was synthesized from sol–gel processing of 4,4′-bis(triethoxysilyl)-1,1′-biphenyl in the presence of the triblock copolymer Pluronic P123. After Soxhlet extraction of the surfactant from the organosilica powder, the material was carbonized at 1000 °C under argon. Elemental analysis of the black solid revealed a carbon content of 45 wt % which can be attributed to free and carbidic carbon species. The initial particle shape according to scanning electron microscopy may be retained after the thermal treatment. TEM, nitrogen sorption measurements, and small angle X-ray scattering indicate that the nanostructure of the final SiOC material is rather similar compared to the organosilica precursor. The surface area and pore volume of the organosilica material of 462 m^2^g^−1^ and 0.5 cm^3^g^−1^, respectively, were only slightly increased to values of 524 m^2^g^−1^ and 0.55 cm^3^g^−1^ for the carbonized sample. The SiOC was thoroughly tested as an anode material in lithium and sodium ion batteries with galvanostatic charge/discharge measurements. In the case of lithium, the material was able to achieve initial discharge capacities of up to 674 mAhg^−1^ at a specific current of 50 mAg^−1^. The mesoporosity also favored higher capacities at significantly higher specific currents, for instance, a capacity as high as 200 mAhg^−1^ could be achieved at 3 Ag^−1^. Promising results were also obtained for sodium. When cycling at a lower current of 50 mAg^−1^, specific capacities of up to 226 mAhg^−1^ could be obtained. When cycling at a higher current, such as 500 mAg^−1^, a capacity of around 100 mAhg^−1^ may still be retained.

In the course of this work, we have shown that mesoporous carbon-rich SiOC shows excellent electrochemical properties which makes it an interesting alternative compared to state-of-the-art anode materials for lithium and sodium ion batteries. The good rate capability can be directly related to the porous nanostructure of the carbon-rich material, which on the one hand allows for a fast ion diffusion through the material and on the other hand helps to accommodate structural integrity, which in turn provides stable cycle performance throughout repeated (de)lithiation/sodiation.

## Figures and Tables

**Table 1:** Nitrogen sorption and SAXS data.

Sample	Nitrogen Sorption			SAXS
	BET Surface Area (m^2^g^−1^)	Pore Volume (cm^3^g^−1^)	BJH Pore Diameter (nm)	d-Spacing (nm)
BPO	462	0.5	4.5 *	4.6
BPO-C	524	0.55	3.5 *	3.8

* BJH pore sizes were determined from the adsorption branch of the isotherms.

**Table 2:** Electrochemical data for BPO and BPO-C at a specific current of 50 mAg^−1^

Alkali Metal	Cut-Off Potentials (V)	1st Cycle		10th Cycle	
		C_extraction_ (mAhg^−1^)	Efficiency (%)	C_extraction_ (mAhg^−1^)	Efficiency (%)
Li	0.005–3	674	50	545	98
Na	0.005–3	226	38	166	97

## Supplementary Materials

The following are available online at https://www.mdpi.com/2079-4991/9/5/754/s1, Figure S1: TEM image of sample BPO. Figure S2: Large TEM image of sample BPO-C.
