# Supplementary material for: Biphenyl-Bridged Organosilica as a Precursor for Mesoporous Silicon Oxycarbide and Its Application in Lithium and Sodium Ion Batteries
Source: Nanomaterials (Basel). 2019 May 16;9(5):754. doi: 10.3390/nano9050754 (PMC6566949; doi:10.3390/nano9050754)
Supplement: Supplementary file 1 [file nanomaterials-09-00754-s001.pdf]

nanomaterials

Supplementary data

Biphenyl-Bridged Organosilica as a Precursor for Mesoporous Silicon Oxycarbide and its Application in Lithium and Sodium Ion Batteries

Manuel Weinberger [1,*], Po-Hua Su [1], Herwig Peterlik [2], Mika Lindén [3], and Margret Wohlfahrt-Mehrens [1,4]

[1] Helmholtz Institute Ulm (HIU), Karlsruher Insitute of Technology, Helmholtzstraße 11, D-89081 Ulm, Germany; surobert82803@gmail.com (P.-H.S.); Margret.Wohlfaht-Mehrens@zsw-bw.de (M.W.M.)
[2] Faculty of Physics, University of Vienna, Boltzmanngasse 5, A-1090 Vienne, Austria; herwig.peterlik@univie.ac.at
[3] Insitute for Inorganic Chemistry II, Ulm University, Albert-Einstein-Allee 11, D-89081 Ulm, Germany; mika.linden@uni-ulm.de
[4] Zentrum für Sonnenenergie-und Wasserstoffforschung (ZSW), Helmholtzstraße 8, D-89081 Ulm, Germany
* Correspondence: manuel.weinberger@kit.edu; Tel.: +49-731-50-34211

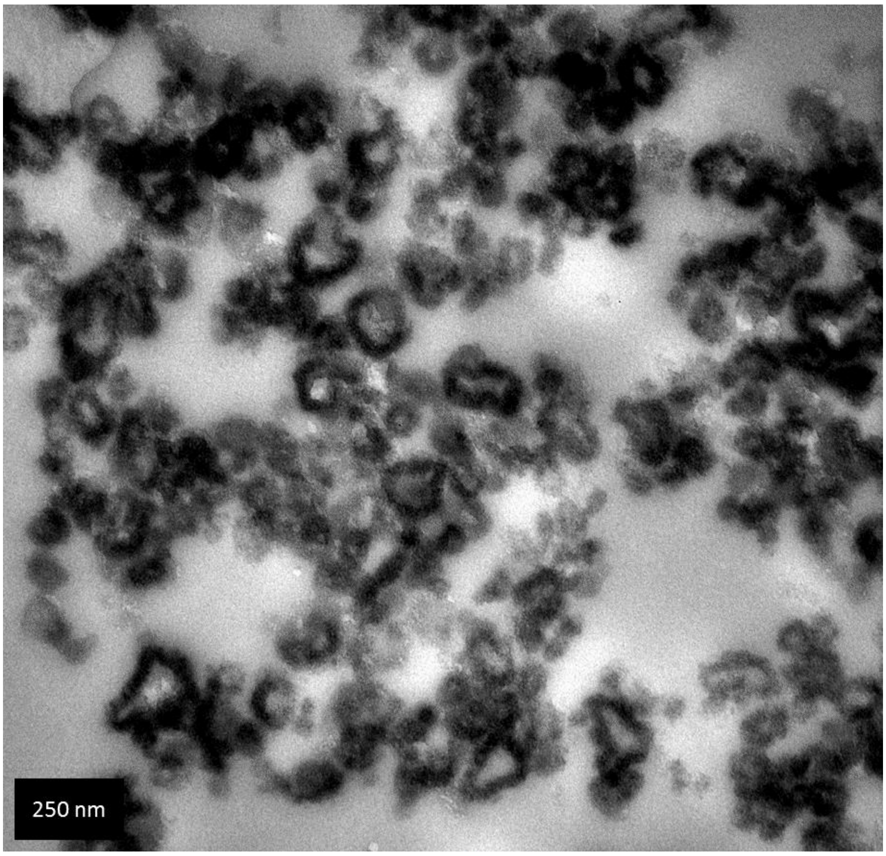

Figure S1. TEM image of sample BPO.

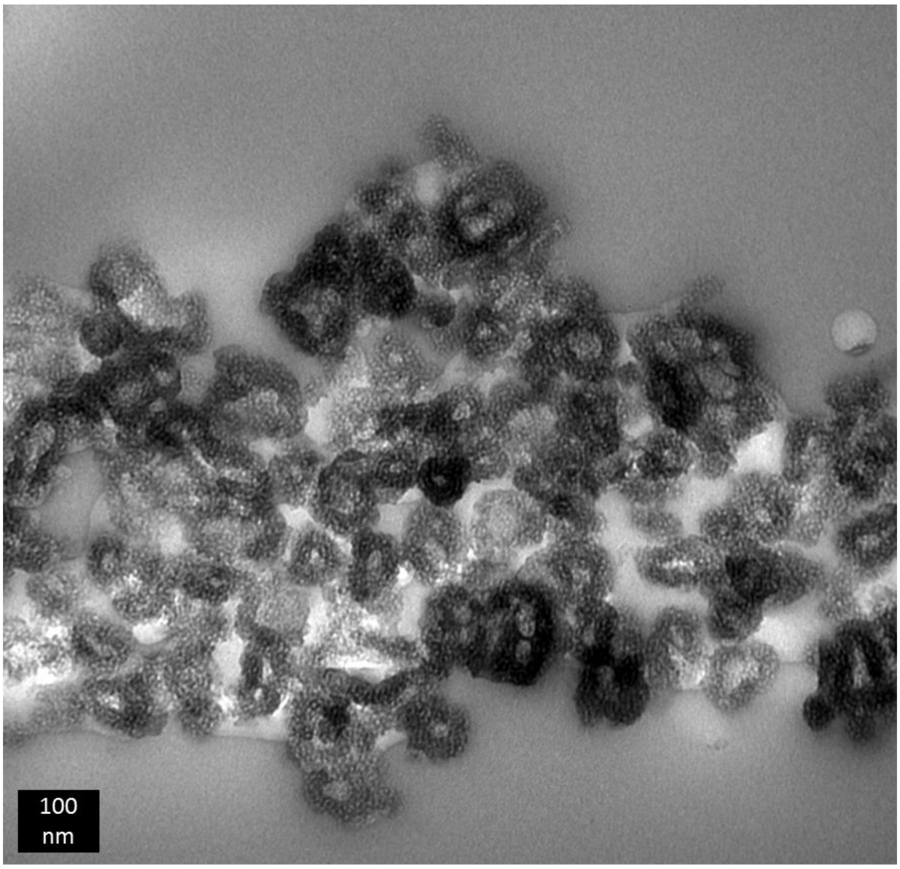

Figure S2. Large TEM image of sample BPO-C.
